# High-Energy Fluidic Microfluidizer Produced Whole Germinant Oat Milk: Effects on Physical Properties and Nutritional Quality

**DOI:** 10.3390/foods13223708

**Published:** 2024-11-20

**Authors:** Qimin Wei, Jun Chen, Taotao Dai, Feiyue Ma, Lizhen Deng, Yingying Ke, Yihui Wang, Laichun Guo, Chunlong Wang, Chao Zhan, Changzhong Ren, Ti Li

**Affiliations:** 1 State Key Laboratory of Food Science and Resources, Nanchang University, Nanchang 330047, China; weiqimin0209@163.com (Q.W.); chen-jun1986@hotmail.com (J.C.); ncubamboo@163.com (T.D.); mafeiyuefenglingcao@126.com (F.M.); denglz1@126.com (L.D.); 357900220002@email.ncu.edu.cn (Y.K.); yhui_wang@163.com (Y.W.); 2 International Institute of Food Innovation Co., Ltd., Nanchang University, Nanchang 330047, China; 3 National Oat Improvement Center, Baicheng Academy of Agricultural Sciences, Baicheng 137000, China; guolaichun@126.com (L.G.); 13500830529@139.com (C.W.); 18204366627@163.com (C.Z.)

**Keywords:** whole oat milk, germination, high-energy fluidic microfluidizer, milk making

## Abstract

Whole oat milk (WOM) was prepared from germinated oat by an innovatively designed high-energy fluidic microfluidizer (HEFM). The results indicated that germination treatment significantly raised the content of total protein, γ-aminobutyric acid, total phenolics, and reducing sugar, while it decreased the content of total starch and β-glucan. Oat with a germination time of 48 h had the best nutritional quality for producing WOM. The physical stability of the WOM prepared from germinated oat was effectively improved by HEFM treatment. The apparent viscosity increased, the instability index reduced from 0.67 to 0.37, and the precipitate weight ratio decreased from 13.54% to 9.51%. As the pressure of the HEFM increased from 0 to 120 MPa, the particle size decreased from 169.5 to 77.0 µm, which was helpful to improve the physical stability of the WOM. Meanwhile, the color of the WOM became whiter after the HEFM treatment. The content of β-glucan and soluble protein in the WOM significantly increased, which was due to the disruption of cells by the HEFM processing. The optimal HEFM pressure for WOM production is 120 MPa. This study provided a new way to produce whole oat milk with a high nutritional quality and good physical properties.

## 1. Introduction

With growing awareness of nutrition and health, consumers are putting increased emphasis on plant-based and whole-grain diets [1]. In the context of more recent studies and market trends, health enthusiasts, environmentalists, lactose-intolerant individuals, and those with specific health and environmental needs can all benefit from plant-based milk. In addition to providing consumers with more options, a variety of whole-grain ingredients have been utilized to produce plant-based milk, such as legumes, nuts, and cereals. Among them, oat milk has become one of the new milk substitutes and developed rapidly because of its delicate taste, easy absorption, high nutritional value, and strong satiety. In addition, oat milk is rich in soluble dietary fiber and phenolic substances, which have physiological functions such as promoting gastrointestinal digestion and lowering cholesterol. However, common crushing methods have a low degree of damage to the cell wall structure of oat, and it is difficult to fully release the bioactive substances in oat milk [2]. Filtration will lead to the loss of oat nutrients, waste oat resources, and reduce the utilization rate of oat [3]. Thus, novel methods of milk production should be developed to enhance the stability of oat milk.

Germination technology is a biotechnology to improve the nutritional content and bioactive substances of cereals [4,5]. It can significantly increase the content of phenols and γ-aminobutyric acid in whole grains [6], which exhibit many positive effects on the prevention and control of hyperlipidemia, hypertension, obesity, and other chronic metabolic syndromes [7,8]. Some research has mainly studied the nutritional changes during the germination process of oats [9,10], but there have been no studies that have used germinated oats as raw materials to prepare whole oat milk. Therefore, in order to raise the nutritional quality of oat milk, germinated oat was innovatively used to prepare whole oat milk in this study.

Ultrafine grinding technology is utilized to modify the physicochemical properties of food products. Our team created a high-energy fluidic microfluidizer (HEFM) system; the structural diagram and working principle have been described in detail in our preliminary research [11]. HEFM technology can disperse and mix the nutrients in food more effectively, making it easier for the body to absorb and use them, while improving the stability of food, extending its shelf life, and reducing food waste. The HEFM used to be called the industry-scale microfluidizer system (ISMS). Based on the demand for processing capacity, the cavity flow channel diameter of the HEFM was expanded from 300 to 700 μm. The ISMS was no longer sufficient to summarize and reflect the current technology and equipment, so our team named it as the HEFM for the first time. The HEFM primarily uses a high-speed current kinetic energy reaction chamber and a high-pressure pump to provide exceptional ultrafine grinding results on samples. Compared to other processing methods, the HEFM can realize filtration-free oat milk, with its full utilization and minimal waste, which is conducive to realizing the utilization of all components of oats and enhancing the physical stability of oat milk. The ISMS has been widely used in the preparation of full-component beverages without filtration. Previous studies have shown that the ISMS has been beneficial to crush cells, reduce particle size, promote nutrient release, and improve physical stability. For example, the ISMS could reduce the particle size of corn pulp and increase the total polyphenol content of whole corn slurry [12]. It has been reported that whole soybean milk prepared by the ISMS without filtration could be self-stabilized for 21 days at 4 °C [11]. The particle size was smaller, the viscosity was higher, and the microstructure of dietary fiber was looser, which improved the stability of the whole soybean milk. The isoflavone content in the whole soybean milk was also increased after ISMS treatment. Dai et al. [13] prepared whole peanut milk using the ISMS. As the treatment pressure increased, its physical stability was significantly improved, and it could maintain no stratification at 4 °C for 67 h. Ke et al. also [14] reported that the application of the ISMS improved the stability of whole mango juice, as well as the effective fragmentation of mango cells to promote the release of nutrients. Therefore, the HEFM has the potential to improve the physical stability and nutritional quality of germinated oat milk, but this has not been reported. We hypothesized that oat milk prepared by the combination of germination and the HEFM would have higher nutrition and good physical stability.

To sum up, in order to solve the problems of low nutrient content and poor stability, germination technology combined with the HEFM were used to produce whole oat milk (WOM). The influences of the germination process on the nutrient content (starch, reducing sugar, β-glucan, γ-aminobutyric acid, total protein, and total phenol) of oat were studied. Then, the HEFM was used to prepare WOM. The effects of the HEFM pressure on physical properties, including the apparent viscosity, physical stability, particle size, color, microstructure, and nutrient content of the WOM, were investigated, and the possible mechanism of the HEFM improving the stability of the WOM was revealed. The results can provide a theoretical basis for the application of germination technology combined with the HEFM in WOM processing.

## 2. Materials and Methods

### 2.1. Materials and Chemicals

Raw oats (Baiyan 20#) were provided by the Baicheng Academy of Agricultural Sciences (Baicheng, Jilin, China). β-glucan standards, total starch kits, Nile blue A, FITC, and fluorescent whitening agent were purchased from Sigma-Aldrich (Shanghai, China). Amylase and saccharifying enzyme were obtained from Novozymes (Bagsvaerd, Copenhagen, Denmark). All other chemicals used in this study were of analytical grade.

### 2.2. Preparation of Germinated Oat

The oats were cleaned and soaked for an entire night. The ratio of oats to water during the soaking was 1:4. Then, the oats were put on trays lined with wet gauze in an incubator at 20 °C for 72 h. During the germination process, water was supplied to the oats as needed. Samples were taken from the germinated oats at 12 h intervals and analyzed immediately. Partial samples were freeze-dried and kept at −20 °C until nutrient analysis. Oats with different germination times were photographed using an optical microscope (CKX41, Olympus Corporation, Tokyo, Japan).

### 2.3. Nutrients Determination of Germinated Oats

The content of reducing sugar was measured using dinitrosalicylic acid (DNS) [15]. The total starch content was determined according to the method of the total starch kit. The protein content of the germinated oats was determined by the Kjeldahl method. The β-glucan content was determined by the method reported by Setyawan et al. [16]. The content of γ-aminobutyric acid in the germinated oats was determined by the method reported by Zhang et al. [17]. The total phenol content of the germinated oats was determined using colorimetry [18].

### 2.4. Preparation of Whole Germinated Oat Milk

The germinated oats with 48 h were treated at a total ratio to water of 1:9 (oat: water, *w*/*v*) and were added into a pre-pulverizer. The crude slurry obtained from the pre-pulverizer (XCFG-2018, Beijing Collaborative Innovation Food Technology Co., Ltd., Beijing, China) was enzymolized using amylase and saccharifying enzyme for 1 h and then treated by the HEFM at 30, 60, 90, and 120 MPa for one pass; the correspondingly produced WOM samples were named WOM_30_, WOM_60_, WOM_90_, and WOM_120_, respectively. The one treated by the pre-pulverizer but without the microfluidizer was named WOM_0_. To prevent microbial growth, 0.02% (*w*/*w*) sodium azide was added into the sample. Partial samples were stored in a refrigerator (BCD-252T, Electrolux Co., Ltd., Wuxi, China) at 4 °C until analysis. Partial freeze-drying was used for subsequent nutrient analysis.

### 2.5. Physicochemical Determination of Whole Germinated Oat Milk

#### 2.5.1. Rheological Properties and Physical Stability

The rheological properties of the WOM samples were determined by the method reported by Li et al. [11], using a rheometer (MCR 302, Anton paar, Graz, Austria). A total of 16 mL of WOM was transferred into a concentric cylinder, and the shear rate ranged from 0.1 to 100 s^−1^ [11].

The precipitate weight ratio was determined using the method of Ke et al. [14]. The WOM samples were centrifuged using a centrifugal machine (LXJ-IIB centrifuge, Shanghai, China) at 4800 g for 20 min. The precipitate weight ratio was calculated as

P = (W_p_/W_m_) × 100 (1)

where W_p_ and W_m_ are the precipitate weights (g) and WOM weights (g), respectively.

The instability index of WOM was performed by using an analytical centrifuge (LUMiFuge, LUM GmbH, Berlin, Germany), based on the method of Ma et al. [19].

#### 2.5.2. Particle Size Distribution (PSD)

The PSD of the WOM was measured using a Mastersizer 3000 (Malvern Instruments Co., Ltd., Worcestershire, UK). The particle refraction index and absorption rate were set to 1.590 and 0.001. The dispersant was deionized water (refractive index of 1.33) [14].

#### 2.5.3. Microscopic Observation

The cell structure of the WOM was observed using an optical microscope (CKX41, Olympus Corporation, Tokyo, Japan). A total of 20 μL of germinated whole oat milk was dropped onto a slide and observed with a 100× lens. The distributions of proteins, starch, and dietary fiber in the WOM samples were observed using CLSM (LSM710, Carl zeiss, Jena, Germany). Nile blue A (1 mg/mL), FITC (1 mg/mL), and fluorescent whitening agent (1 mg/mL) were mixed with the WOM at the ratios of 1:50 (*v*/*v*), 1:60 (*v*/*v*), and 1:50 (*v*/*v*), respectively. Microscope images were acquired using three laser excitation sources (488, 405, and 633 nm) and appropriate emission channels.

#### 2.5.4. Color

The WOM samples were measured using a colorimeter (CM-5, Konica Minolta, Tokyo, Japan). The values of L*, a*, and b* were recorded. The color difference was calculated from the data:

(2) $$\Delta E=\sqrt{{\left(L_{0}^{*}-L^{*}\right)}^{2}-{\left(a_{0}^{*}-a^{*}\right)}^{2}+{\left(b_{0}^{*}-b^{*}\right)}^{2}}$$

The WOM_0_ sample was used as a reference.

#### 2.5.5. Nutrients

The determination of the β-glucan and total phenolic content was the same as the method in Section 2.3. The soluble protein content was measured based on the method of Jiang et al. [3]. Totals of 5 mL of the samples were centrifuged at 4000× *g* rpm for 20 min, and then 500 μL of the supernatant was dyed with Coomassie blue G250 solution. The absorbance was measured at 595 nm. And, bovine serum albumin was used as a standard protein.

### 2.6. Statistical Analysis

All experiments were conducted three times. Data mapping was performed using Origin 2021 (OriginLab Co., Northampton, MA, USA). Statistical analysis was performed using SPSS 26.0 (SPSS Inc., Chicago, IL, USA). All data were analyzed using one-way ANOVA and a post hoc Duncan’s test for multiple comparisons. Results were evaluated using the Tukey test (*p* < 0.05) and expressed as the mean ± standard deviation.

## 3. Results and Discussion

### 3.1. Appearance of Germinated Oats and Nutrients Content

#### 3.1.1. Appearance

**Figure 1:**
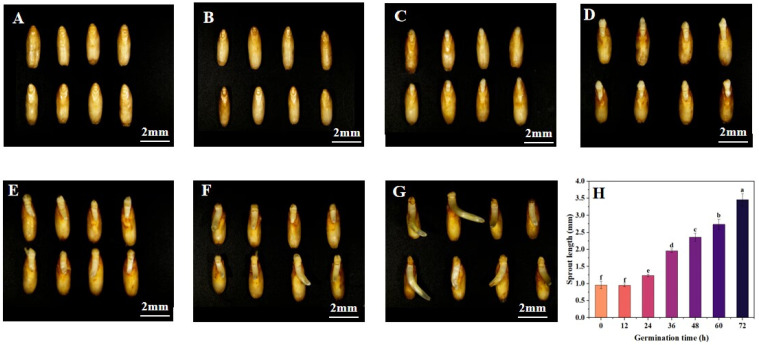
The morphology of oats for different germination times of 0 h (**A**), 12 h (**B**), 24 h (**C**), 36 h (**D**), 48 h (**E**), 60 h (**F**), 72 h (**G**) and sprout length in different germination times (**H**). Note: Different letters indicate significant (*p* < 0.05) differences in the sprout length for different germination times.

As shown in Figure 1, the growth of the oat buds increased with the prolongation of the oat germination time, and there was a significant difference in the oat length at different germination times. The bud length of the oat reached the maximum value of 3.458 mm after germination for 72 h. The results indicated that the oat could germinate and grow normally under a constant temperature and humidity, which could provide energy and material exchange basis for subsequent growth.

#### 3.1.2. Starch and Reducing Sugar

**Figure 2:**
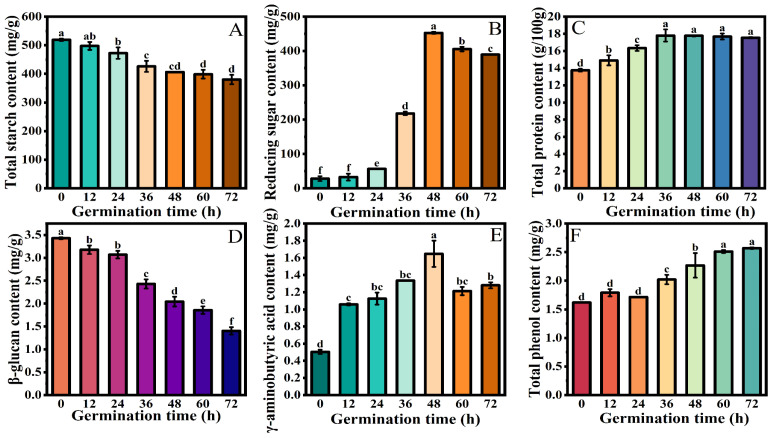
Changes in nutrient content of oats at different germination times: (**A**) total starch, (**B**) reducing sugars, (**C**) total protein, (**D**) β-glucan, (**E**) γ-aminobutyric acid, (**F**) total phenolic content. Note: Different letters indicate significant (*p* < 0.05) differences in above nutritional indexes of oats treated with different germination.

Starch, the primary ingredient in oats, has a significant impact on the texture and processing of oat products. As shown in Figure 2A, the starch content of the raw oat was 518.97 mg/g. Compared to the ungerminated oats, the starch content of the germinated oats decreased with the extension of the germination time. After germination for 36 h, the starch content was 426.28 mg/g, which was decreased by 17.86%. This may be due to the fact that during the soaking process, oat seeds were puffed out after absorption, and hydrolases (α-amylase, β-amylase, etc.) were activated. Under the action of enzymes, starch is decomposed into small-molecular sugars to provide energy for the growth of oat [20]. With the germination time being too long, the activity of starch-related enzymes decreased, and the decrease rate of the starch content decreased. The effect of different germination times on reducing sugar contents in the oat can be seen in Figure 2B. With the germination time increasing, the content of reducing sugar in the oat first increased and then decreased. After germination for 48 h, the content of reducing sugar reached its highest (452.79 mg/g). The enzymatic breakdown of starch into small-molecular sugars during oat germination may be the cause of the increase in reducing sugar content during germination [21]. With the germination time further increasing, the decrease in the reducing sugar content may be caused by the energy decomposition for the synthesis of new substances and the germination of the oat.

#### 3.1.3. Protein Content

The change in the protein content of the oats for different germination times is shown in Figure 2C. With the germination time increasing, the protein content of the oats first increased and then stabilized, and the protein content of the ungerminated oats was the lowest (13.75%). After germination for 12 h, the protein content increased, and it was the highest at 17.8% after germination for 36 h. After continuing to prolong the germination time, there was no significant change in the protein content. Benincasa et al. [8] also found that the protein content of germinated oats was higher than that of raw oats. The increase in protein content during germination may be due to the dry weight loss of oats in the form of carbohydrates through respiration, resulting in changes in nitrogenous substances during germinating [10].

#### 3.1.4. β-Glucan Content

As the main water-soluble macromolecular polysaccharide in oat, β-glucan is mostly distributed in the aleurone layer of oat grains. It makes up a significant portion of the plant seeds’ endosperm cell wall [22]. As shown in Figure 2D, with the extension of the germination time, the β-glucan content showed a significant decreasing trend, and that in the ungerminated oats was the highest (3.43 mg/g). The β-glucan content in the oat decreased after germination for 12 h. The β-glucan content of the oat reached its lowest at 1.36 mg/g after germination for 72 h, which was decreased by 151%. This may be due to the activation of β-glucanase during oat germination, which leads to the hydrolysis of β-glucan and a decrease in β-glucan content. Yoshimura et al. [23] also found that the β-glucan content of barley decreased after germination treatment.

#### 3.1.5. γ-Aminobutyric Acid Content

As an inhibitory neurotransmitter, γ-aminobutyric acid plays a key role in the human central system, controlling elevated blood pressure, reducing anxiety, and stimulating insulin secretion [24,25]. Figure 2E indicates that the content of γ-aminobutyric acid increased and then decreased as the germination time increased. The γ-aminobutyric acid content was significantly increased from 0.50 mg/g to 1.06 mg/g, an increase of 0.55 times (*p* < 0.05) that of the raw oats after germination for 12 h. The content increased after the germination time was extended to 48 h. Its peak value was 1.65 mg/g, which was 3.27 times that of the non-germinated group. Çelik et al. [26] found that the content of γ-aminobutyric acid in germinated oats was significantly increased. This may be because the germination activated the activity of glutamate decarboxylase, accelerated the decarboxylation of glutamate, and increased the production of γ-aminobutyric acid. As the germination time increased to 60 and 72 h, the γ-aminobutyric acid content was significantly decreased. This may be due to the inhibition of γ-aminobutyric acid transaminase activity, which converts γ-aminobutyric acid to succinic semi-aldehyde, resulting in a large amount of γ-aminobutyric acid consumption [27].

#### 3.1.6. Total Polyphenol Content

Research results have shown that the long-term consumption of whole grains and related products can help reduce the incidence of chronic diseases, which is related to the content of polyphenols contained in whole grains [28]. In Figure 2F, it is shown that the total polyphenol content of the oat increased significantly with the extension of the germination time and then tended to be stable. The content was the highest (2.57 mg/g) when the germination time reached 72 h. These findings are consistent with those in a germinated millet, with a significant increase in the total polyphenol content [28,29]. The increases in the total polyphenol content may be because the soaking process softened the oat seeds, making it easier to extract the partially bound phenols bound to cell wall polysaccharides. On the other hand, upon germination, oat anthranilamide synthase activity increases, and oats produce a considerable amount of anthranilamides [29].

In conclusion, germination treatment can improve the nutritional quality of raw oat. The contents of reducing sugar, total protein, aminobutyric acid, and total phenol in the oat were the highest after germination for 48 h. Germinated oat was used for the subsequent preparation of whole oat milk.

### 3.2. Effect of HEFM Treatment on Physicochemical Properties of WOM

#### 3.2.1. Rheological Properties

**Figure 3:**
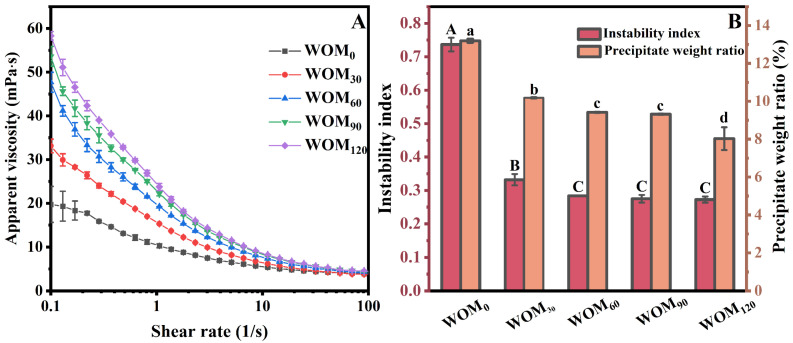
Effect of different HEFM processing pressures on apparent viscosity (**A**) and precipitate weight ratio and instability index (**B**) of germinated whole oat milk. Note: Different letters indicate significant (*p* < 0.05) differences in precipitate weight ratio and instability index of germinated whole oat milk.

The rheological properties of beverages are important parameters for beverages and play a vital role in the optimization of beverage processing. Proper viscosity makes a beverage more stable and provides a richer and fuller taste [12,30]. Non-Newtonian pseudoplastic behavior is of great significance for both consumers and product formulators, which can not only enhance the performance and usability of products but also provide fresh perspectives and methodologies for the creation of innovative offerings. This, in turn, delivers a greater consumption experience to consumers and elevates overall satisfaction. Figure 3A shows the rheological characteristics of WOM treated with the HEFM. The apparent viscosity of the WOM dropped as the shear rate increased, showing that the WOM had the non-Newtonian characteristics of a pseudoplastic fluid. As the HEFM treatment pressure increased, the apparent viscosity of the WOM progressively increased. This could be because the HEFM produced a strong shear force and high-speed impact force, thus increasing the content of soluble substances in the WOM [31]. Simultaneously, the HEFM altered the internal components of the system or enhanced the interactions between the substances, which resulted in an increase in the apparent viscosity of the WOM. The improvement in viscosity could alleviate the gravitational subsidence of particles to a certain extent, thus improving the stability of the WOM [3].

#### 3.2.2. Instability Index and Precipitate Weight Ratio

As shown in Figure 3B, the instability index of the oat milk decreased significantly after the HEFM treatment. The instability index of the WOM_0_ was 0.73, and that of the WOM_60_ was 0.28. But there was no significant difference in the instability index of the oat milk as the treatment pressure continued to increase. The decreases in the instability index may be due to the fact that a large number of larger particles in the WOM were crushed into smaller particles after the HEFM treatment, delaying the sedimentation rate of the particles. The precipitation weight ratio is one of the indicators to assess the stability of beverages. The precipitate weight ratio of the WOM prepared by the HEFM under different pressures was determined to study its effect on the stability of the oat milk. With the increase in the HEFM treatment pressure, the precipitate weight ratio of the oat milk gradually decreased, and that of the sample with a treatment at 120 MPa was the lowest, which decreased by 64.66% compared to the WOM_0_.

#### 3.2.3. Particle Size Distribution

**Figure 4:**
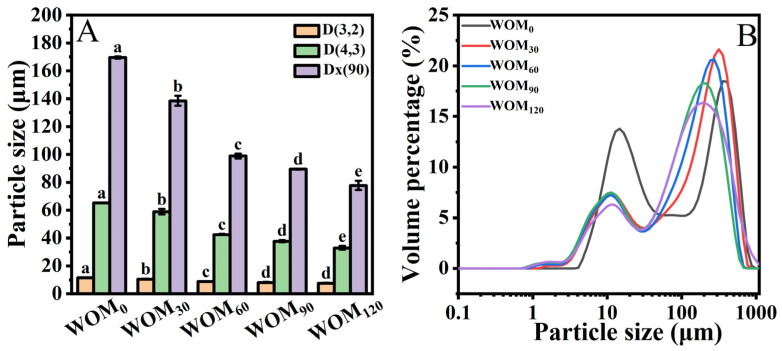
Effect of different HEFM processing pressures on particle size (**A**) and distribution (**B**) of whole germinated oat milk. Note: Different letters indicate significant (*p* < 0.05) differences on particle size of germinated whole oat milk.

Particle size plays an important role in the stability of a beverage. In terms of effects on sensory parameters like texture and mouthfeel, particle size reduction is important to consider for both researchers and food industry practitioners. The particle size and distribution of the WOM prepared by the HEFM with different pressures were measured. As shown in Figure 4A, the particle size of the WOM decreased with the increase in the HEFM treatment pressure. The D (4,3) and D (3,2) of the WOM_0_ were the highest, which were 62.55 and 11.5 μm, respectively. Compared to the WOM_0_, the D (4,3) and D (3,2) of the WOM_120_ decreased, respectively, by 90.41% and 52.11%. The effects of different HEFM pressure treatments on the particle size distribution of the WOM are shown in Figure 4B. The particles ranging from 1 to 50 µm were mainly oil bodies and protein aggregates, while the particles ranging from 50 to 300 µm were mainly composed of dietary fiber, starch, and particle aggregates [32,33]. With the increase in the HEFM treatment pressure, the particle size distribution of the WOM shifts to the left. It indicates that the HEFM treatment had a good crushing effect on large particles such as protein, dietary fiber, and starch [34,35]. It could significantly reduce the particle size of the WOM, which was conducive to delaying the particle settlement in the WOM and making the system more stable. This may be due to the strong shear and impact forces during the HEFM treatment. Also, the high-intensity machining provided a greater crushing energy [12].

#### 3.2.4. Microstructure of WOM

**Figure 5:**
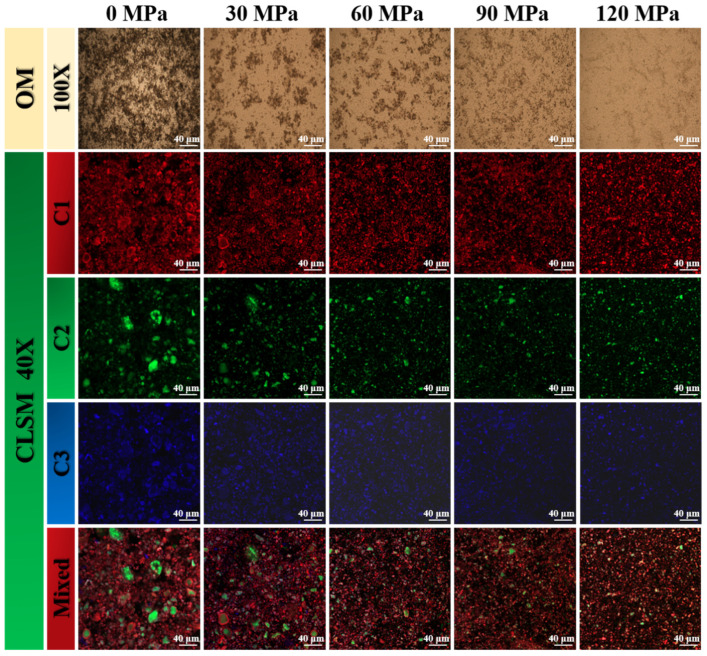
Effect of different HEFM processing pressures on CLSM and optical microscopy images of whole germinated oat milk. Note: Cl 1 is the protein in the oat milk, C2 is the starch in the oat milk, C3 is the dietary fiber in the oat milk, and the mixing channel is the mixing diagram of the channels.

The effects of the HEFM treatment on the morphology of the WOM are shown in Figure 5. A large number of unbroken cell structures were observed in the WOM_0_, and large starch granules contained in the cells would have led to the instability and precipitation of the oat milk. The same cell wall structure could also be observed in the 30 MPa samples, but it had been fragmented and the amount of it was reduced, which may have be caused by the cell structure destruction of the HEFM. Scattered fibrous structures were also observed in the 60 MPa samples and gradually decreased or disappeared in the 90 and 120 MPa samples, indicating that the fiber cells and cell walls in the WOM were crushed. The CLSM results of the HEFM treatment on the whole oat milk are also shown in Figure 5. The protein in the samples of 0 and 30 MPa showed irregular large particles. With the continuous increase in treatment pressure, large protein particles were cracked and soluble protein gradually penetrated the WOM system. This may be because the strong impact of the HEFM destroyed the protein structure and transformed the large protein particles into small proteins. The changes in the starch particles and dietary fiber were similar to those of the protein; the starch and dietary fiber became smaller as the treatment pressure increased and were uniformly dispersed in the oat milk system. Optical microscopy and CLSM showed that the HEFM treatment could effectively break macromolecular substances such as protein, starch, and dietary fiber in the oat milk and significantly reduce the particle size of the oat milk, which was conducive to the stability of the WOM. This can be linked to the results of particle size analysis. The result is similar to that of Li et al. [11] for whole soybean milk and Dai et al. [13] for whole peanut milk.

#### 3.2.5. Color of WOM

**Figure 6:**
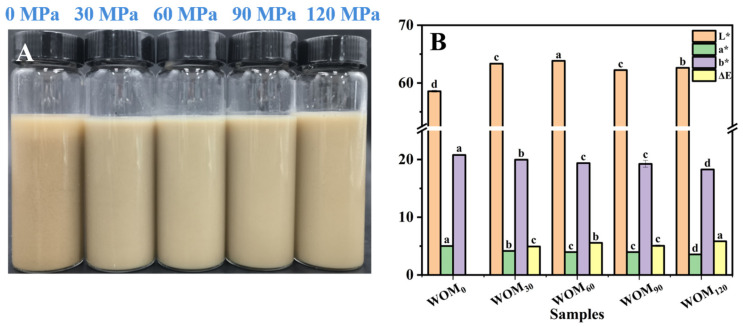
(**A**) Appearance of whole germinated oat milk at different treatment pressures of HEFM. (**B**) Effect of different HEFM processing pressures on color of whole germinated oat milk. Note: different letters represent significant difference between L*, a*, and b* of whole germinated oat milk (*p* < 0.05).

Color is one of the key factors influencing whether customers buy a product or not. For plant protein beverages, milky white is a more appealing color for WOM. Figure 6 shows the color of the WOM changing with the HEFM treatment. The WOM treated with the HEFM had a decreased red-green value (a*) and an increased brightness (L*). Its color was brighter and whiter, and its yellow color was lower. This may be because the HEFM created high pressure, which led to the emulsification of the proteins and the oil droplets in the WOM. Guo et al. [12] reported that the ISMS did not adversely affect the overall appearance of WCS, with the treated product still having a pleasant yellow color. Overall, the HEFM did not adversely affect the overall appearance of the WOM, and the processed product still had a pleasant milky white color. The HEFM process changes the composition and structure of WOM, thus affecting the optical properties of WOM, which plays an important role in its consumer acceptability and desirability.

#### 3.2.6. Nutrients Content

**Figure 7:**
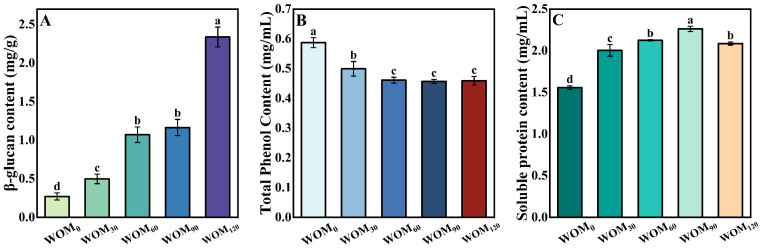
Effect of different HEFM processing pressures on content of whole germinated oat milk: (**A**) β-glucan content; (**B**) total phenol content; (**C**) soluble protein content. Note: Different letters indicate significant (*p* < 0.05) differences in β-glucan, total phenol, and soluble protein content of whole germinated oat milk.

The effects of different HEFM pressures on the β-glucan content in the germinated WOM are shown in Figure 7A. The β-glucan content in the WOM rose with the increase in the HEFM treatment pressure, which reached the highest value when the treatment pressure increased to 120 MPa. It was 2.34 mg/g, which was 8.67 times higher than that in the WOM_0._ This may be because the high-speed impact force and strong shear force during the HEFM treatment caused the oat cell walls to be crushed, and more β-glucan was dissolved from the cell walls [36]. As shown in Figure 7B, the total polyphenol content in the oat milk decreased with the increase in the treatment pressure. The polyphenol content of the WOM_0_ was the highest (0.59 mg/mL). Compared to the WOM_0_, the polyphenol content of the WOM_120_ decreased by 23%. This may have been due to the cavitation vortex effect produced by the huge pressure difference, which changed the dissolved state of oxygen in HEFM in the WOM and accelerated the oxidation reaction [37,38]. It may also have been because of the high temperature generated by the HEFM, which accelerated the loss of polyphenols and structural breakdown of polyphenol compound junctions [37,38]. Figure 7C shows the impact of various HEFM treatment pressures on the content of soluble protein in the WOM. As the treatment pressure increased, the amount of soluble protein in the WOM first increased and subsequently declined. The soluble protein in the WOM_90_ reached a maximum of 2.26 mg/mL, which increased by 45.21% compared to that of the WOM_0_. This increase in soluble protein content may be attributed to the increased degree of cellular fragmentation, dissolving more proteins. When the pressure increased to 120 MPa, the amount of soluble protein decreased. This was due to high temperature produced by the high pressure destroyed the protein structure through processes such as oxidation, denaturation, or disintegration [39]. The findings of this investigation are consistent with those of research on whole-grain oat pulp [3]. Based on the specific changes in all of the above indicators, 120 MPa should be selected as the best process parameter for the preparation of WOM, which could maintain the stability of the system.

## 4. Conclusions

In this study, whole oat milk with rich nutrition and good stability was innovatively prepared by germination and the HEFM without filtration. The results indicated that the contents of reducing sugar and γ-aminobutyric acid in the oat first increased and then decreased, the total polyphenol content increased, and the contents of β-glucan and starch decreased significantly with the increasing germination time. The oat with 48 h of germination had the best nutritional quality and was suitable for oat milk production. After HEFM treatment, the whiteness, β-glucan, and soluble protein content of the WOM increased. The HEFM treatment could effectively reduce the particle size, increase the apparent viscosity of the WOM, enhance the stability of the system, and delay the particle settlement. And, the optimal HEFM pressure for WOM production is 120 MPa. The results of this study showed that germination combined with HEFM treatment can improve the nutrient content and stability of oat milk. It provided a theoretical basis and technical support for the development of whole oat milk and oat products with a higher level of nutrients and good physical properties. And, we will explore the different germination times and HEFM parameters of different grains and plant-based milks in subsequent studies.

## Data Availability

The original contributions presented in the study are included in the article, further inquiries can be directed to the corresponding authors.
